# Continuous aerosol monitoring and comparison of aerosol exposure based on smoke dispersion distance and concentrations during oxygenation therapy

**DOI:** 10.1038/s41598-023-42909-1

**Published:** 2023-09-23

**Authors:** Chih-Chieh Wu, Wei-Lun Chen, Cheng-Wei Tseng, Yung-Cheng Su, Hsin-Ling Chen, Chun-Lung Lin, Tzu-Yao Hung

**Affiliations:** 1 Department of Emergency Medicine, Zhong-Xing Branch, Taipei City Hospital, Taipei, Taiwan; 2 School of Medicine, Tzu Chi University, Hualien County, Hualien, Taiwan; 3 Department of Emergency, Ditmanson Medical Foundation, Chiayi Christian Hospital, Chiayi County, Chiayi, Taiwan; 4 School of Medicine, National Yang-Ming Chiao Tung University, Taipei, Taiwan; 5 CrazyatLAB (Critical Airway Training Laboratory), Taipei, Taiwan

**Keywords:** Diseases, Health occupations, Medical research

## Abstract

This study evaluated the aerosol exposure risks while using common noninvasive oxygenation devices. A simulated mannequin was designed to breathe at a minute ventilation of 20 L/min and used the following oxygen-therapy devices: nasal cannula oxygenation (NCO) at 4 and 15 L/min, nonrebreathing mask (NRM) at 15 L/min, simple mask at 6 L/min, combination of NCO at 15 L/min and NRM at 15 L/min, high-flow nasal cannula (HFNC) at 50 L/min, and flush rate NRM. Two-dimension of the dispersion distance and the aerosol concentrations were measured at head, trunk, and foot around the mannequin for over 10 min. HFNC and flush-rate NRM yielded the longest dispersion distance and highest aerosol concentrations over the three sites of the mannequin than the other oxygenation devices and should use with caution. For flow rates of < 15 L/min, oxygenation devices with mask-like effects, such as NRM or NCO with NRM, decreased aerosol dispersion more effectively than NCO alone or a simple mask. In the upright position, the foot area exhibited the highest aerosol concentration regardless of the oxygenation device than the head–trunk areas of the mannequin. Healthcare workers should be alert even at the foot side of the patient while administering oxygenation therapy.

## Introduction

The severe acute respiratory syndrome coronavirus 2 (SARS-CoV-2) is transmitted via upper-airway discharges, such as sneezing, coughing, or breathing^1,2^. Particularly the continual air flow generated by the airway during breathing can widely distribute the discharges^3^. The discharge particle size ranges from droplets to aerosols, and the number of virions in the particle increases with particle size^4^. SARS-CoV-2 can survive in the aerosol form for ≥ 3 h^5^. Several hospital procedures involving airways may discharge contaminated aerosols (aerosol-generating procedures, AGPs)^6^, including nasogastric tube insertion, oxygenation therapy, noninvasive ventilation, mechanical ventilation, and tracheal intubation^7,8^.

Hypoxia is a symptom of severe COVID-19^9^ for which supplemental oxygen is often administered using multiple devices^10^. Conventional non-invasive oxygen supplementation therapies usually involve low flow rates of < 15 L/min, including nasal cannula oxygenation (NCO; 1–6 L/min), simple mask (5–10 L/min), and non-rebreathing mask (NRM; 10–15 L/min), to provide a fraction of the inspired O_2_ (FiO_2_; 0.24–0.66)^11,12^. Besides conventional O_2_ therapy, a higher flow rate of NCO (6–15 L/min) can increase FiO_2_ from 0.49 to 0.72^13^. More severe hypoxemia requires invasive mechanical ventilation, and preoxygenation is considered an adjunct of O_2_ supplementation before emergent intubation. NCO combined with NRM at 15 L/min can reduce hypoxemia incidence before and during intubation^14,15^. Furthermore, flush-rate NRM exhibits the same efficacy as a bag-valve-mask (BVM) device, becoming widely used for preoxygenation before intubation^16,17^. Ultimately, employing a high-flow nasal cannula (HFNC) reduces the intubation rate among patients with severe hypoxemia. However, it can also potentially introduce significant delays in the intubation process, which subsequently becomes a point of debate^12,18, 19^.

On the other hand, these oxygenation therapies have the potential to disperse contaminated airborne aerosols and droplets, which can linger in the surrounding environment, particularly in situations involving close contact or within enclosed spaces like hospitals^5–7, 20, 21^. Increasing the flow rate of O_2_ devices, such as HFNC, has the potential to enhance FiO_2_ levels, thus potentially improving hypoxia. However, this action could also lead to the dispersion of aerosols and droplets^21,22^. Moreover, the use of personal protective equipment (PPE) may hinder medical interventions, potentially increasing the risk of aerosol exposure due to prolonged tracheal intubation times or reduced performance in endotracheal intubation procedures^23,24^. As a result, achieving a delicate balance between maintaining the safety of medical staff and ensuring effective patient management remains a topic of significant debate.

This quantitative study aimed to evaluate the risk of aerosol exposure by assessing aerosol concentrations at the head, trunk, and feet of a mannequin during various oxygen therapy methods. The study involved a continuous assessment period of 10 min, which provides more reliable insights for medical personnel concerning potential latent infectious risks during treatment. In contrast to previous qualitative aerosol studies with unspecified observation periods^25,26^, our study comprehensively addresses this gap. While our experience suggests that measuring the maximum distance is optimal within the first minute, it's important to note that this may not fully represent the reality of continuous oxygen therapy.

## Methods

### Study design and setting

**Figure 1:**
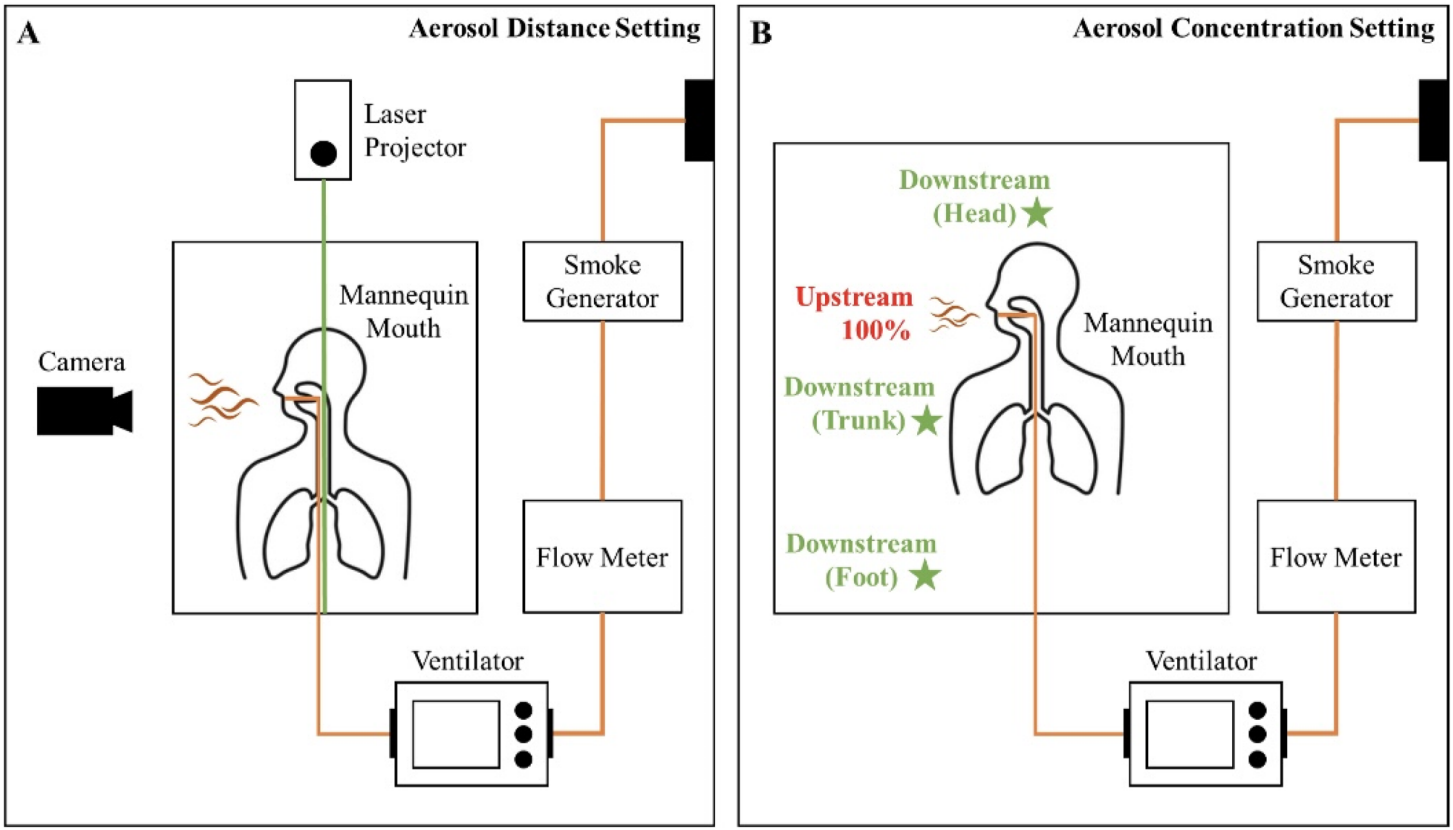
(**A**) Illustrates the experimental setup for measuring aerosol distance. A fixed breathing cycle was provided to the simulation mannequin through a ventilator connected to a smoke generator and air supplementation system. Green lasers were positioned at the foot and head areas for two-dimensional aerosol particle assessment. High-sensitivity cameras captured the dispersion of atomized tracer gas, subsequently analyzed using particle image velocimetry. (**B**) Demonstrates the experimental arrangement for measuring aerosol concentration. The ventilator, smoke generator, and simulation mannequin were used, with air being drawn out during the breathing cycle. Atomized poly-alpha-olefin (PAO) was employed as a tracer gas and released from the mannequin’s mouth. Aerosol concentration detection was conducted with an upstream detector placed close to the mannequin's mouth and downstream detectors at three locations (head, trunk, and foot). Downstream aerosol concentrations were measured and compared with the upstream concentration.

**Figure 2:**
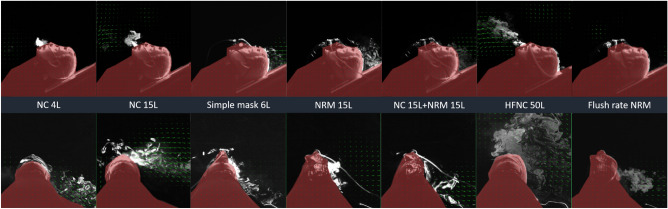
Particle image velocimetry revealed the aerosol movement using a velocity vector (green arrows). The columns from left to right correspond to NC 4 L, NC 15 L, simple mask 6 L, NRM 15 L, NC 15 L + NRM 15 L, HFNC 50 L, and flush-rate NRM, respectively. The upper and lower rows present the results obtained in the sagittal and coronal planes, respectively. Because the mannequin was placed in an inclined head-up 30° position, the aerosol movement in the NC and HFNC groups was mainly dispersed to the foot area of the mannequin. In the NRM, NC 15 L + NRM 15 L, and simple mask groups, the amount of aerosol was decreased, and the aerosols dispersed to the top of the head of the mannequin rather than to the foot area. *NC* nasal cannula, *NRM* nonrebreather mask, *HFNC* high-flow nasal cannula.

This study was conducted within a negative-pressure resuscitation room at the Zhong-Xing branch of Taipei City Hospital, which was specifically designated for COVID-19 treatment in northern Taiwan. The room maintained a rate of 12 air changes per hour. Background air was drawn from the upper region of the space and circulated downward through four vents located at each corner. Within this setup, an Airway Management Trainer (Laerdal) simulation mannequin was positioned in a 30° inclined head-up posture, with its artificial trachea connected to a three-dimensional-printed ventilator (Massachusetts Institute of Technology Emergency Ventilator, Massachusetts, USA). This ventilator was further linked to a smoke particle generator (MPL-I003, Tong-Da, Tainan, Taiwan), which dispersed atomized poly-alpha-olefin (PAO) particles with a diameter of 0.5–0.7 μm, simulating the release of small particles from the mannequin's mouth. The breathing cycle was set at 30 breaths per minute, achieving a minute ventilation of approximately 20 L/min to replicate tachypnea conditions. The ratio of inspiration time to expiration time (I/E ratio) was measured to be 1:1.05. Peak inspiratory and expiratory flow rates were recorded as 20.56 and 22.62 L/min, respectively. To capture the light scattering induced by the tracer gas, a high-sensitivity camera (ORCA-Flash 4.0 v2 digital CMOS camera; Hamamatsu Co., Hamamatsu, Japan) was positioned at a distance of 2 m from the mannequin's mouth. The subsequent analysis utilized the large-area particle image velocimetry (PIV) technique to monitor the dynamic alterations in aerosol distribution during the breathing cycle (depicted in Fig. 1A). PIV facilitated the evaluation of aerosol movement via velocity vectors (illustrated in Fig. 2). Additionally, a scattering photometer was strategically placed in three distinct regions around the mannequin—head, trunk, and foot—to assess the concentration of contaminated aerosols potentially exposing healthcare workers (HCWs) (demonstrated in Fig. 1B).

### Intervention

Seven oxygenation devices were applied to the simulated mannequin: NCO (GREEN CROSS YJ-66, Changshu Taining Medical Equipment Co. Ltd, China) at flow rates of 4 L/min and 15 L/min; NRM (E-UP, Ningbo Shengyurui Medical Appliances Co., Ltd., China) at 15 L/min; simple mask (FOR CARE Enterprise Co., Ltd., Taiwan) at 6 L/min; NCO combined with NRM, both at 15 L/min; HFNC using a Humidoflo^®^ HF-2970 system (Great Group Medical Co., Ltd., Taiwan) at 50 L/min with FiO_2_ 99% ± 4.975%; and flush-rate NRM at 50 L/min. These oxygenation devices were positioned on the mannequin’s face as in clinical practice and compared with the control (no oxygenation device).

### Measurements

Background flow field concentration was initially recorded at the beginning of the experiment. Detection began when PAO concentration fell below < 0.002% (background level). Light-scattering sensors operated continuously at three mannequin positions (head, trunk, foot), recording aerosol concentrations. Maximum aerosol-dispersion length and width were continually measured, with 100 readings taken in a 10-min interval for both control and study groups. An additional 1500 continuous measurements captured aerosol concentrations at the mannequin's head, trunk, and foot in both control and study groups.

### Outcomes

The primary outcome of this study was the aerosol-dispersion length and width while using the oxygenation devices. The secondary outcome was the aerosol concentrations at the mannequin’s head, trunk, and foot for each device vs. the reference group. The aerosol-dispersion flow fields visualized while using the devices were also assessed for correlation with the aerosol-dispersion distances and concentrations.

### Statistical analysis

Student’s *t*-test was performed to evaluate the mean percentage differences among the oxygen device settings. Statistical analyses were performed using SPSS, version 29.

## Results

The aerosol-dispersion distance (length and width) from the oxygenation devices was measured via the particle velocity technique (Table 1). The mean dispersion length and width of the aerosols with no oxygenation devices was 0.56 ± 0.09 and 0.41 ± 0.05 m, respectively; this was considered the reference group. For HFNC, the mean length and width of the aerosol-dispersion distance was 1.35 ± 0.10 and 1.01 ± 0.20 m, respectively, which were the longest among the seven methods and significantly differed from the reference group. During NRM at 15 L/min, the mean length and width of the aerosol-dispersion distance was 0.79 ± 0.04 and 0.26 ± 0.05 m, respectively, which were the shortest distances of all the settings. The mean distances (length and width) of the remaining two settings were 1.05 ± 0.05 and 0.71 ± 0.14 m for NCO combined with NRM and 1.26 ± 0.10 and 0.64 ± 0.13 m for flush-rate NRM, respectively; these two settings exhibited significant differences with the reference group. The mean length and width of the aerosol-dispersion distance of a simple mask at 6 L/min was 1.10 ± 0.11 and 0.94 ± 0.19 m, respectively, with the length of the dispersion alone being significantly different from the reference group (Table 1).

**Table 1:** The aerosol dispersion distance (both in length and width) for the seven oxygenation devices was assessed using the particle velocity technique. The dispersion distance refers to the extent of aerosol spread from the mannequin's mouth and is measured in meters (m). We measured and compared the mean, standard deviation, mean difference, and 95% confidence interval (CI) of the dispersion distance for each device with that of the no oxygenation therapy (used as a reference). Significance was established at a threshold of P ≤ 0.001. *CI* confidence interval, *NC* nasal cannula, *NRM* nonrebreather mask, *HFNC* high-flow nasal cannula.

Dispersion length					Dispersion width			
Mean (m)	Standard deviation	95% CI	P value		Mean (m)	Standard deviation	95% CI	P value
0.56	0.09	0.54–0.58	–	Reference	0.41	0.05	0.40–0.43	–
1.17	0.14	1.15–1.20	< 0.001	NC 4L	0.81	0.29	0.75–0.87	< 0.001
1.17	0.09	1.15–1.19	< 0.001	NC 15L	0.94	0.19	0.90–0.98	< 0.001
1.10	0.11	1.08–1.12	< 0.001	Simple mask 6L	0.94	0.19	0.90–0.98	< 0.001
0.79	0.04	0.78–0.80	< 0.001	NRM 15L	0.26	0.05	0.25–0.27	< 0.001
1.05	0.05	1.04–1.06	< 0.001	NC 15L + NRM 15L	0.71	0.14	0.68–0.74	< 0.001
1.35	0.10	1.33–1.37	< 0.001	HFNC 50L	1.01	0.20	0.97–1.05	< 0.001
1.26	0.10	1.24–1.28	< 0.001	Flush rate NRM	0.64	0.13	0.61–0.67	< 0.001

**Figure 3:**
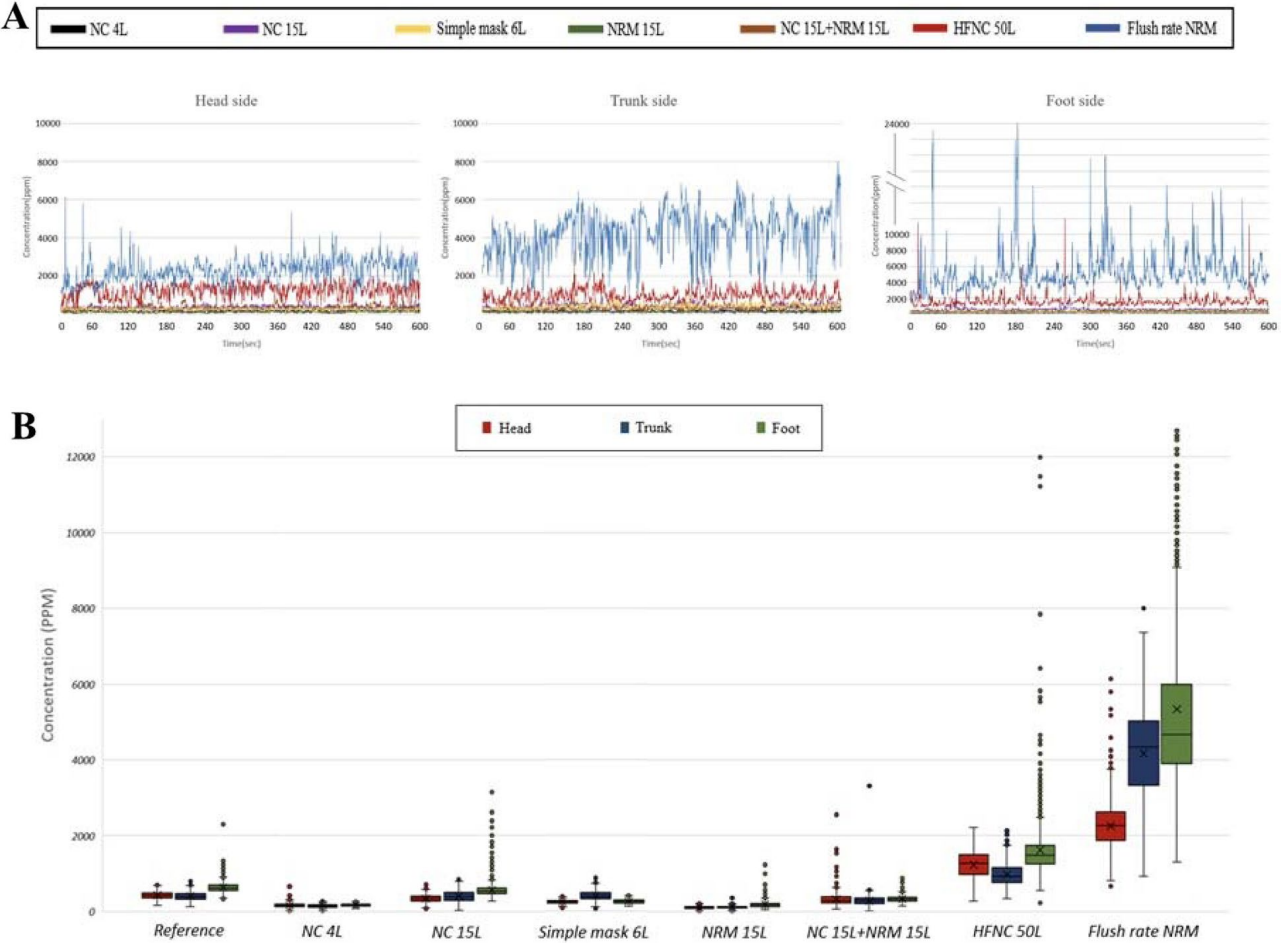
(**A**) Aerosol concentrations across seven oxygenation settings over 10 min. The colored lines represent aerosol concentrations for different settings: black (NC 4 L), purple (NC 15 L), brown (NC 15 L + NRM 15 L), red (HFNC 50 L), green (NRM 15 L), blue (flush-rate NRM), and yellow (simple mask 6 L). The vertical axis shows particle concentration (parts per million, ppm, 10–6), while the horizontal axis represents time (s). Aerosol concentrations at the head, trunk, and foot areas are displayed in the left, middle, and right columns, respectively. *PPM* parts per million (10^–6^), *NC* nasal cannula, *NRM* nonrebreather mask, *HFNC* high-flow nasal cannula. (**B**) Box plot of aerosol concentrations under eight oxygenation settings, including seven oxygenation-delivering devices and the reference group (no oxygenation), recorded at the head, trunk, and foot areas. The red, blue, and green columns correspond to the head, trunk, and foot areas, respectively. The box's top represents the 75th percentile, the bottom represents the 25th percentile, the line in the middle signifies the 50th percentile, and crosses indicate mean values. Whiskers indicate outliers.

The aerosol concentrations in each group were recorded from 0 to 10 min, yielding 1500 samples. The data were defined as proportions by comparing the downstream aerosol concentrations (head, trunk, or foot) with upstream aerosol concentrations (mannequin mouth, defined as 100%) (Fig. 3B). The mean, standard deviation, mean difference, and 95% confidence interval (CI) of the aerosol concentrations of the seven oxygenation devices at the head, trunk, and foot areas were compared with those of the reference group (Table 2). The mean aerosol concentrations in the reference group at the head, trunk, and foot areas were 428.52 ± 100.10, 407.12 ± 118.80, and 631.95 ± 137.40 ppm, respectively (*P* < 0.001). Boxplots and line graphs of the study are provided in Fig. 3B. The mean concentration at the three detection sites was the highest in the flush-rate NRM group at 2261.91 ± 601.75, 4171.45 ± 1192.61, and 5342.02 ± 2603.87 ppm at the head, trunk, and foot, respectively (*P* < 0.001). The HFNC group exhibited mean particle concentrations of 1233.12 ± 354.29, 980.14 ± 312.57, and 1626.50 ± 765.67 ppm at the head, trunk, and foot, respectively (*P* < 0.001) (Table 2).

**Table 2:** Mean, standard deviation, mean difference, and 95% confidence interval of the aerosol concentrations afforded by the seven oxygenation devices were measured and compared with the reference group (without oxygenation therapy) at the head, trunk, and foot areas of the mannequin within a 10-min period. The mannequin’s mouth was defined as 100% aerosol concentration. Significance was set at *P* ≤ 0.005. *ppm* parts per million (10^–6^), *CI* confidence interval, *NC* nasal cannula, *NRM* nonrebreather mask, *HFNC* high-flow nasal cannula.

		Mean (ppm)	Standard deviation	Mean difference	95% CI	P value
Head	Reference	428.52	100.10	–	–	–
	NC 4L	159.48	56.66	− 269.04	156.61–162.35	< 0.001
	NC 15L	338.49	96.44	− 90.03	333.61–343.38	< 0.001
	Simple mask 6L	243.15	51.76	− 185.37	240.53–245.78	< 0.001
	NRM 15L	106.08	37.86	− 322.44	104.16–108	< 0.001
	NC 15L + NRM 15L	322.93	165.08	− 105.59	314.57–331.29	< 0.001
	HFNC 50L	1233.10	354.29	804.58	1215.17–1251.10	< 0.001
	Flush rate NRM	2261.90	601.75	1833.38	2231.44–2292.40	< 0.001
Trunk	Reference	407.12	118.80	–	–	–
	NC 4L	142.05	43.82	− 265.07	139.83–144.27	< 0.001
	NC 15L	401.93	140.34	− 5.19	394.82–409.04	0.275
	Simple mask 6L	404.91	120.24	− 2.21	398.82–411.00	0.613
	NRM 15L	111.87	36.16	− 295.25	110.04–113.70	< 0.001
	NC 15L + NRM 15L	286.00	126.36	− 121.12	279.60–292.40	< 0.001
	HFNC 50L	980.14	312.57	573.02	964.31–995.97	< 0.001
	Flush rate NRM	4171.50	1192.60	3764.38	4111.05–4231.90	< 0.001
FOOT	Reference	631.95	137.34	–	–	–
	NC 4L	170.02	34.99	− 461.93	168.25–171.79	< 0.001
	NC 15L	563.99	200.32	− 67.96	553.85–574.14	< 0.001
	Simple mask 6L	254.79	49.989	− 377.16	252.26–257.33	< 0.001
	NRM 15L	185.55	94.384	− 446.40	180.772–190.33	< 0.001
	NC 15L + NRM 15L	329.68	78.671	− 302.27	325.69–333.67	< 0.001
	HFNC 50L	1626.50	765.67	994.55	1587.72–1665.30	< 0.001
	Flush rate NRM	5342.00	2603.90	4710.05	5210.15–5473.90	< 0.001

Moreover, besides HFNC and flush-rate NRM groups, the mean concentration of the reference group was higher than those of the remaining five groups (*P* < 0.001) (Table 2). The concentration timeframes and the boxplots revealed numerous outliers around the HFNC and flush-rate NRM groups, suggesting the turbulent spread of aerosols (Fig. 3A,B). Furthermore, the foot aerosol concentrations were substantially higher in the flush-rate NRM group (95% CI 5210.14–5473.90 ppm) compared with the HFNC group (95% CI 1587.71–1665.27 ppm) (Fig. 3, Table 2). Generally, the concentrations in each group were higher at the foot vs. the head area (Table 2).

## Discussion

The aerosols generated by breathing primarily exhibit sizes below 1 μm, with the majority falling between 0.5 to 1 μm^25,26^. It is important to note that the SARS-CoV-2 virus can remain viable in aerosols for a duration of approximately 1.1 to 1.2 h^5^. In our simulation study, we employed tracer gas particles with a diameter of 0.5–0.7 μm (and a median aerodynamic diameter, MMAD, of 0.62 ± 0.16 μm)—a measurement that is nearly comparable to, albeit slightly smaller than, previous research findings^25,26^. Despite this size difference, the behavior and movement of these particles within the flow field demonstrate remarkably similar comparative characteristics.

Numerous studies have documented aerosol dispersions from various oxygen-delivery devices^27–29^. Hung et al.^29^ reported maximum aerosol dispersion at different minute ventilation settings recorded within 3 min: 51.49 ± 19.47 cm for the reference group (no oxygen device), 64.31 ± 14.39 cm for HFNC 30 L/min, 67.09 ± 12.74 cm for HFNC 70 L/min, 85.55 ± 7.28 cm for NCO 15 L/min, and 63.08 ± 15.33 cm for NRM 15 L/min at 20 L/min. Hui et al.^27^ also reported aerosol-dispersion distances for NCO at 1, 3, and 5 L/min of < 42 cm, while those for NRM at 6, 8, 10, and 12 L/min were < 10 cm. In our study, spanning a 10-min observation period, we found an aerosol dispersion of NCO at 4 L/min to be 117.17 ± 14.26 cm in length and 80.71 ± 29.48 cm in width. Interestingly, despite the lower flow rate used in Hui's study, both Hung's and our studies exhibited larger aerosol dispersion lengths and widths compared to the 3-min and 10-min periods, implying a time-dependent increase in aerosol dispersion for the reference group (no oxygenation therapy) and NCO. The aerosol dispersion of NRM, although increased with time, remained the lowest among the non-invasive oxygen therapy methods. During COVID-19 outbreak, HFNC was considered the most effective approach for hypoxia as it decreased the intubation rate in patients with respiratory failure^18,19^. However, the risk of aerosol transmission while using this method remains unknown^19,21, 22^. Hui et al.^28^ reported aerosol-dispersion values of 17.2 ± 3.3, 13.0 ± 1.1, and 6.5 ± 1.5 cm in normal lungs for HFNC at 60, 30, and 10 L/min, respectively. In our study, the length and width of aerosol dispersion in HFNC were the longest. Following a 10-min observation, the aerosol dispersion of HFNC 50 L/min was 1.35 ± 0.10 m in length and 1.01 ± 0.20 m in width. In contrast, in Hung’s study of HFNC with flow rates of 70 L/min, the maximum dispersion was 1.00 ± 0.01 m in length and 0.57 ± 0.02 m in width. This finding suggests that Hui’s study may have observed a shorter period than both the 3-min period in Hung's study and the 10-min period in our study^29^. Overall, the aerosol dispersion distance of HFNC remained the largest among the non-invasive oxygenation devices studied^27–29^.

HFNC involves an air/oxygen blender, active humidifier, single heated tube, and nasal cannula. It delivers adequately heated and humidified medical gas at a flow of 0–70 L/min, affording several physiological advantages than other standard oxygen therapies, including reduced anatomical dead space, upper airway positive end-expiratory pressure, constant FIO_2_, and good humidification. Moreover, it decreases the breathing frequency and work, thereby reducing intubation requirement in patients with COVID-19^18,19^. In severe hypoxia, escalating respiratory support requirement should be considered alongside oxygenation, which increases oxygen reserves to prevent hypoxemia during apnea before intubation. Driver et al.^16^ reported that airway oxygenation using flush-rate NRM provided an airflow of 50–54 L/min, which was noninferior to that of BVM devices. Herein, flush-rate NRM yielded the highest aerosol concentration compared with HFNC and the remaining oxygenation devices (Fig. 3A,B). Notably, this contrasted with the aerosol-dispersion distance, as a greater distance did not reflect a higher concentration. Unsealed interface around the airway and oxygen devices may explain these findings as aerosol leak more from the flush-rate NRM than in HFNC. The visible condensed aerosols can be more easily captured via the high-sensitivity camera, however, the invisible accumulated aerosol concentration may reflect the aerosol exposure more authentically.

In patients with mild-to-moderate hypoxia, oxygen can be delivered using NCO or simple masks with an oxygen flow of approximately 4–6 L O_2_/min followed by titration of the flow rates by monitoring pulse oximetry, aiming for an oxyhemoglobin saturation on pulse oximetry (SpO_2_ > 88%)^30^. In all the study groups, the aerosol concentrations were higher in the foot area vs. the trunk or head areas (Table 2). Gravity affected the aerosols exhaled from the mouth of an upright mannequin at a 30-degree angle, which, according to projectile motion, exhibited a parabolic trajectory. With decreasing kinetic energy, the aerosols remained suspended at the foot area, thus accumulating and showing increased concentration (Fig. 3). Hung et al.^29^ reported that aerosol concentration during NRM at 15 L/min was higher in the head area vs. the foot and trunk areas in a 3-min observation period; owing to the holes on the upper part of the mask, the particle concentration in the head area rapidly increased. Conversely, this study was conducted over 10 min, which could explain this difference. We assumed that the aerosol was exhaled from the mannequin’s mouth toward the foot area; therefore, the aerosol concentrations were significantly higher in the foot area vs. other sites regardless of the oxygenation device, especially in the HFNC and flush-rate NRM groups (Table 2). Additional studies are necessary to evaluate the effects of aerosols according to the patient’s inclination angle.

NRM at 15 L/min and NCO at 4 L/min yielded the lowest aerosol concentration in the three areas (Table 2). Increasing the NCO flow to 15 L/min also resulted in an increase in aerosol concentrations. Moreover, the oxygenation devices that use masks (NRM at 15 L/min, NCO at 15 L/min combined with NRM at 15 L/min, and simple mask at 6 L/min) yielded lower particle concentration levels than other devices (Table 2). Compared with NCO and HFNC, the oxygen mask covers the mouth and nose, and the air leaking from the gap between the edge of the mask and the face flows easily toward the ground in round-shaped mask settings. As the mask in NRM seals more tightly than a simple mask, the former causes less aerosol leaks, resulting in lower aerosol concentrations. The aerosol concentrations in NCO combined with NRM at 15 L/min were lower than those of NCO alone at 15 L/min in the three areas (Table 2). Therefore, except at a flow rate ≥ 15 L/min, nasal cannula devices with poorer sealing place HCWs at a higher risk of exposure to contaminated aerosols compared to oxygen mask devices.

### Limitations

This simulation study might not perfectly replicate real clinical conditions. The chosen fixed ventilation rate of 20 L/min was intended to simulate a desaturated patient with breathlessness, and PAO particles were released as traceable aerosols. However, it may not precisely mirror lung physiology due to various physiological factors, such as respiratory irregularities, depth, droplet distribution, tidal volume, and virion deactivation rates, which are influenced by factors like droplet radius, temperature, and humidity. Nonetheless, the standardized experimental setup allowed us to delineate aerosol dispersion and exposure among the seven common oxygenation devices for the study's purpose. Additionally, it's important to note that this study did not account for scenarios where the mannequin wears a face mask, which might not accurately reflect real-life conditions where patients wear masks.

## Conclusion

The aerosol concentrations were lower when the flow rate of the non-invasive oxygenation devices was < 15 L/min or in the presence of mask effects, such as NRM and NCO with NRM. However, the aerosol concentrations increased in high-flow oxygenation devices (i.e., HFNC and flush-rate NRM), which surpassed the mask effect, further increasing the aerosol contamination levels and risk of infection among HCWs. Furthermore, the aerosol concentrations were higher in the foot area vs. the trunk and head areas for all the oxygenation devices; thus, HCWs should be more cautious around the foot area if the oxygenation therapy is applied to the patient in an upright position with an aerosol transmissible disease.

### Supplementary Information

Supplementary Information.

## Data Availability

All data generated or analyzed during this study are included in this published article and its supplementary information files.
