# Author Correction: Continuous aerosol monitoring and comparison of aerosol exposure based on smoke dispersion distance and concentrations during oxygenation therapy

**DOI:** 10.1038/s41598-023-47564-0

**Published:** 2023-11-22

**Authors:** Chih‑Chieh Wu, Wei‑Lun Chen, Cheng‑Wei Tseng, Yung‑Cheng Su, Hsin‑Ling Chen, Chun-Lung Lin, Tzu‑Yao Hung

**Affiliations:** 1 Department of Emergency Medicine, Zhong‑Xing Branch, Taipei City Hospital, Taipei, Taiwan; 2 School of Medicine, Tzu Chi University, Hualien County, Hualien, Taiwan; 3 Department of Emergency, Ditmanson Medical Foundation, Chiayi Christian Hospital, Chiayi County, Chiayi Taiwan; 4 School of Medicine, National Yang-Ming Chiao Tung University, Taipei, Taiwan; 5 CrazyatLAB (Critical Airway Training Laboratory), Taipei, Taiwan

Correction to: *Scientific Reports* https://doi.org/10.1038/s41598-023-42909-1, published online 23 September 2023.

The original version of this Article contained an error in the spelling of the author Chun-Lung Lin, which was incorrectly given as Chung-Lung Lin.

The original Article has been corrected.

